# Optimum temperature may be a misleading parameter in enzyme characterization and application

**DOI:** 10.1371/journal.pone.0212977

**Published:** 2019-02-22

**Authors:** Vitor M. Almeida, Sandro R. Marana

**Affiliations:** Departamento de Bioquímica, Instituto de Química, Universidade de São Paulo, São Paulo, São Paulo, Brazil; Shantou University Medical College, CHINA

## Abstract

The optimum temperature is commonly determined in enzyme characterization. A search in the PubMed database for “optimum temperature” and “enzymes” yielded more than 1,700 manuscripts reporting this parameter over the last five years. Here, we show that the optimum temperature is not a constant. The catalytic activity of the mesophylic β-glucosidase Sfβgly was determined at different temperatures using different assay times and enzyme concentrations. We observed that the optimum temperature for Sfβgly changed by 5°C simply by modifying the assay length, and it was inversely correlated with enzyme concentration. These observations rely on the fact that close to the melting temperature, thermal denaturation continuously decreases the active enzyme concentration as the assay progresses. Thus, as the denaturation rate increases with increasing temperature, the bell-shaped curves observed in “activity *versus* temperature plots” occur only if the enzyme is denatured at and above the optimum temperature, which was confirmed using the thermostable β-glucosidase bglTm. Thus, the optimum temperature hardly reflects an intrinsic enzyme property and is actually a mere consequence of the assay condition. Thus, adoption of the “optimum temperature” determined under bench conditions for large-scale uses, which differ in assay length and enzyme concentration, may result in lower yields and financial losses.

## Introduction

Enzymes have been employed as biotechnological tools for decades. The first patent reporting the utilization of an enzyme was registered in 1894 [1]. Recently, enzyme applications have increased, including use in the production of biofuels, as detergent additives, in sewage treatment, in textile industry processes, in the pulp and paper industry and to improve food and beverage quality [1]. The enzyme world market was estimated at USD 5 billion in 2016 [2]. In addition, enzymes are the main targets of medicines, a market which was worth approximately USD 160 billion in 2017 [1, 2].

Naturally, the economic and health importance of enzymes prompts intense interest in enzyme production and characterization.

The optimum temperature is commonly determined in enzyme characterizations. For example, a search for “optimum temperature” and “enzymes” in the PubMed database returned 232 manuscripts published in 2017. In the last five years, more than 1,700 manuscripts have reported this parameter. Despite its broad use, the optimum temperature is not actually a constant that reflects an intrinsic enzyme property, as previously discussed in fundamental enzymology textbooks [1, 3].

In an attempt to call attention to this contradiction and its potential pitfalls, we present an objective experimental demonstration of the fleeting nature of the optimum temperature by using two homologous enzymes, a mesophilic (Sfβgly from *Spodoptera frugiperda*; UniProt O61594) and a thermostable (bglTm *Thermatoga maritima*; UniProt Q08638) β-glucosidase.

In brief, we show that the optimum temperature is a relative term that depends on the assay duration and protein concentration employed in enzyme characterization. Due to this dependence on conditions that easily change between characterization studies and large-scale applications, the blind use of the optimum temperature may result in financial and efficiency losses. Therefore, this work extends beyond a mere academic issue and may contribute to the efficient biotechnological use of enzymes.

## Material and Methods

### Expression and purification of Sfβgly and bglTm

The production and purification of Sfβgly and bglTm were performed as previously described [4, 5]. Briefly, DNA encoding for Sfβgly and bglTm proteins was separately cloned into the plasmids pET46 and pLate51, respectively, which were then transformed into *E*. *coli* BL21 (DE3) cells using heat shock (30 min on ice, 40 s at 42°C, then 5 min on ice). Cells with recombinant vectors were cultured at 37°C and 200 rpm in 500 mL of rich medium (Luria Broth) containing antibiotics (50 μg/mL ampicillin for bacteria containing pLate51 vector and 100 μg/mL carbenicillin for those with pET46) until an optical density at 600 nm of 0.6 was reached. Afterward, expression of the recombinant protein was induced by adding 1 mM isopropyl β-thio-galactopyranoside (IPTG) and incubating the bacteria for 24 h at 20°C and 200 rpm. Bacterial cells were then centrifuged at 7,000 x*g* and 4°C for 30 min. Pellets were resuspended in 5 mL of lysis buffer (10 mM sodium phosphate buffer, pH 7, with 100 mM NaCl and 20 mM imidazole) and disrupted by sonication using 5 12-s pulses at an output of 3 in a Branson Sonifier 250 (Branson Instruments, Stanford, CT, US). After another round of centrifugation with the same conditions, the supernatant was collected and gently mixed with nickel nitriloacetic resin (Qiagen, Valencia, CA, US) (200 μL of resin for each 1 mL of supernatant) for 30 min at 6°C. The contaminant proteins adsorbed in the resin were removed by washing with lysis buffer (10 cycles of resuspension and centrifugation). The recombinant proteins specifically bound to the resin were eluted with 10 mM sodium phosphate buffer at pH 7 containing 100 mM NaCl and 500 mM imidazole. After elution, the purified proteins were transferred to 100 mM citrate-phosphate buffer at pH 6 using High Trap Desalting Columns (GE HealthCare, Little Chalfont, UK). The purity of the samples was analysed via SDS-PAGE [6] (S1 Fig).

### Optimum temperature assay

The enzymatic activities of Sfβgly and bglTm were detected with the substrate *p*-nitrophenyl β-D-glucopyranoside (NPβGlc; 6 mM) using the absorbance at 415 nm following the release of *p*-nitrophenolate. This substrate concentration corresponded to ten-fold the *K*_m_ of NPβGlc [5, 7]. The substrates and enzymes were prepared in 100 mM citrate-phosphate buffer at pH 6. The assays were performed at 29, 33, 37, 42 and 46°C for at least 100 min. The enzymatic reaction was stopped every 10 min with the addition of 250 mM sodium carbonate at pH 11. The experimental product *versus* time data were fitted into the linear equation [P]_t_ = [P]_0_ + *v*t when the assay temperatures were between 29 and 37°C and into the exponential equation [P]_t_ = [P]_0_ + A*e*^kt^ when the assay temperatures were 42 and 46°C using Origin 8.0 software (OriginLab Corporation; Northampton, USA) (S2 Fig). [P]_t_ is the product concentration at time t, and [P]_0_ is the initial product concentration. In the linear equation, the term “*v*” is the initial reaction rate, which is directly proportional to the enzyme activity (μmol/min, i.e., U). In the exponential equation, the term “A” is a pre-exponential factor, and “k” is the [P] increment constant. The enzymatic activity at different time points of the [P] *versus* t curves was calculated based on the first-order derivative determined using the Origin 8.0 software. In the linear curves, the first derivative is equal to the slope “*v*”. Deviations of the first derivative, i.e. enzymatic activity, were calculated based on 3 assays at each temperature (S2 Fig). Errors of the relative activities (S1–S3 Tables) were calculated by propagation of the first derivative deviations using the equation c = (a^2^ + b^2^)^1/2^, where “a” and “b” are the percentual deviations of two first derivatives and “c” is the percentual error of their relative activity [8].

## Results and discussion

**Fig 1:**
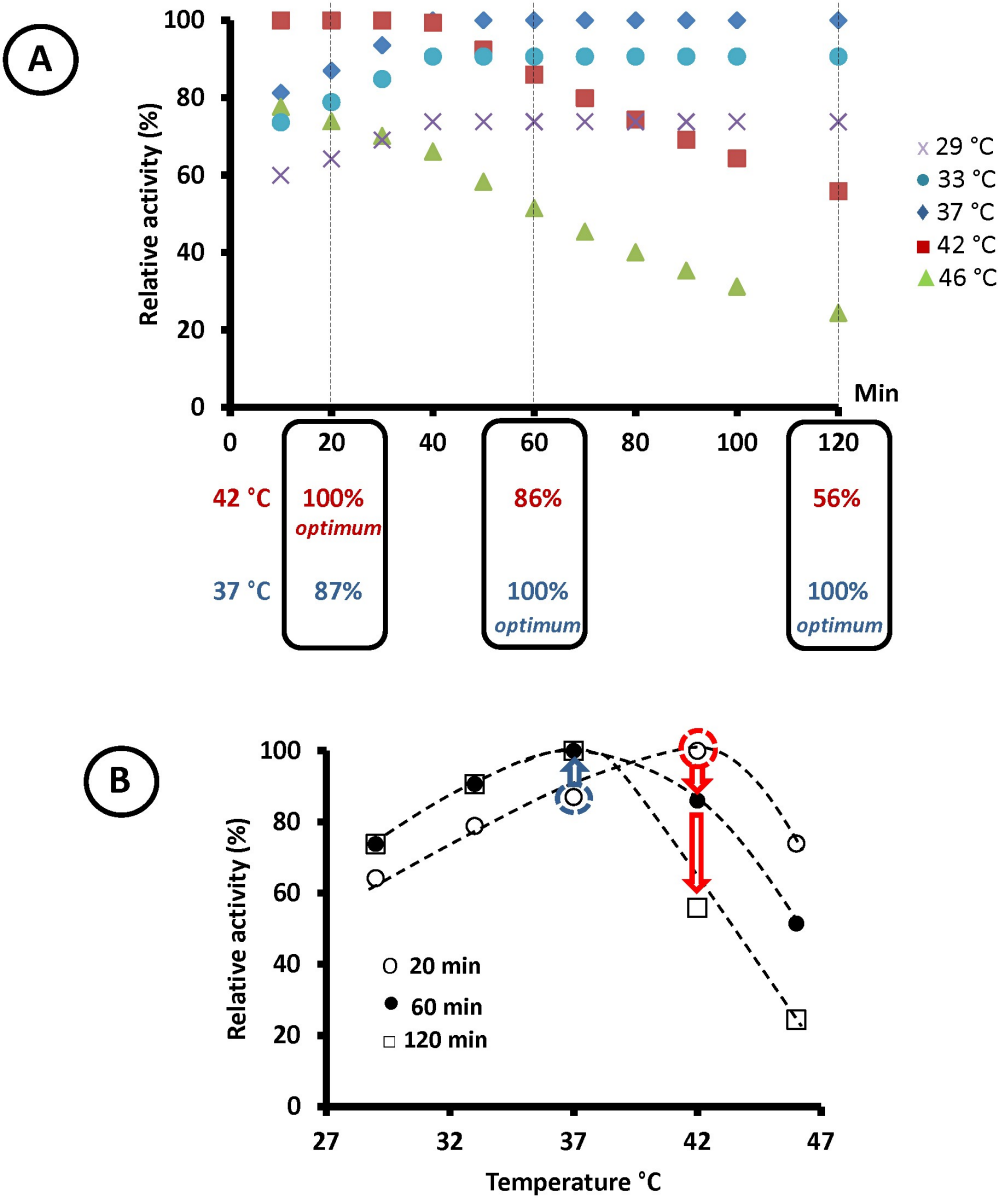
Effect of assay duration on the optimum enzyme temperature. **A)** Relative activity of Sfβgly during the assays at different temperatures. (purple cross) 29°C; (blue circle) 33°C; (blue diamond) 37°C; (red square) 42°C; (green triangle) 46°C. Vertical dashed lines highlight three different assay times (20, 60 and 120 min). The relative activity at 42 and 37°C at these assay times is presented in the rounded boxes, illustrating the shift of the optimum temperature throughout the assay. **B)** Changes in the optimum temperature resulting from modifications of the enzyme assay duration. The red circle and arrows highlight the decrease in the enzyme activity at 42°C, which was the optimum temperature in the shorter assay. The blue circle and arrow illustrate the increase in the relative activity at 37°C, which was the optimum temperature only for longer assay times. The enzyme concentration was 140 nM. This complete dataset (mean relative activities and respective deviations) is presented on S1 Table.

To demonstrate that the optimum enzyme temperature depends on the assay conditions, the β-glucosidase Sfβgly was submitted to the “classic” procedure for optimum temperature determination, i.e., its activity was determined at different temperatures (29 to 46 °C) using the same enzyme concentration. Then, the Sfβgly activity during the course of a fixed-temperature assay was determined using the first derivative at specific points of the product *versus* time curve (S2 Fig). Based on these results, the relative activity of Sfβgly was calculated at distinct times (10 to 120 min), which made possible the examination of how the optimum temperature plots evolved during the assays of the same enzyme (Fig 1).

An overview of Fig 1 reveals that the relative position of the activity data associated with each temperature changes with assay time (Fig 1A). For example, at 20 min, the highest relative activity, i.e., the optimum temperature, was observed at 42 °C. Conversely, at 60 min, the optimum temperature was 37 °C. Thus, the optimum temperature changed by 5 °C by modifying the assay length, while the remaining conditions (enzyme and substrate concentrations and buffer) were constant. More dramatically, in the 120-min assay, the relative activity at 42 °C—the former optimum temperature—dropped to only 50%. Therefore, plots of the temperature effect on the relative enzyme activity for different assay durations clearly show different shapes and maxima, i.e., optimum temperatures, even when produced from the same enzyme (Fig 1B).

**Fig 2:**
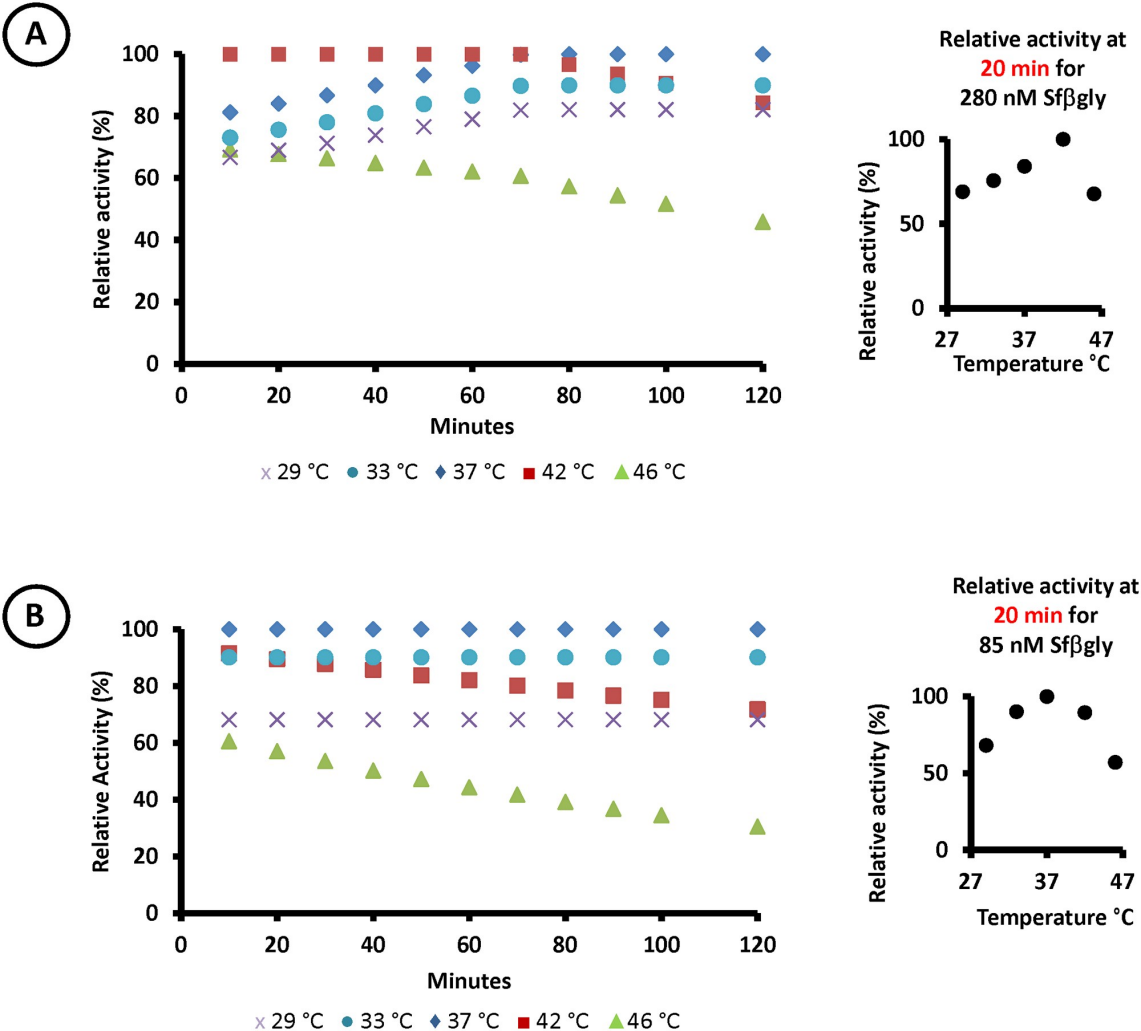
Effect of the enzyme concentration on the optimum temperature. **A)** Relative activity of Sfβgly during the assays at different temperatures in experiments performed using 280 nM Sfβgly and **B)** 85 nM Sfβgly. (purple cross) 29°C; (blue circle) 33°C; (blue diamond) 37°C; (red square) 42°C; (green triangle) 46°C. Inserts show the optimum temperature plot for 20-min assays at each enzyme concentration. Comparison of the inserted plots illustrates the optimum temperature shift due to enzyme dilution. This complete dataset (mean relative activities and respective deviations) is presented on S2 Table.

As an additional demonstration that the optimum temperature is not a constant parameter, the experiment described above was repeated by employing two different concentrations of Sfβgly (Fig 2). It is clear that the relative positioning of the activity data evolved differently during the course of the assay for the experiments performed with 85 and 280 nM Sfβgly. The plots of the temperature effect on enzyme activity prepared with data from 20-min assays indicated that the optimum temperature for 280 nM Sfβgly was 42 °C (Fig 2A), whereas the optimum temperature for this same enzyme at 85 nM was 37 °C (Fig 2B). Therefore, the optimum temperature was also affected by the enzyme concentration.

In conclusion, the optimum temperature changed with modification of the assay time and enzyme concentration. Thus, it is not a parameter that reflects an intrinsic enzyme property but is instead a mere consequence of the assay conditions.

The molecular basis of the observed modifications of relative activity and optimum temperature relies on the fact that the enzyme population is not at thermodynamic equilibrium in the “classic procedure” for optimum temperature estimation. Briefly, at temperatures close to and above the enzyme melting temperature (*T*_m_), the active enzyme concentration continuously decreases over the course of the assay due to thermal denaturation of the protein. The rate of protein denaturation is much lower below the *T*_m_, so the concentration of the active enzyme does not change in this temperature range. Additionally, the higher the temperature, the larger the fraction of the substrate population that reaches the transition state, which increases the reaction rate. These trends are simultaneous throughout the enzyme activity assay. Thus, at temperatures that allow the decrease in enzyme activity caused by protein denaturation to overcome the reaction rate increase caused by temperature, the detected enzyme activity drops during the course of the assay. In contrast, at temperatures at which no protein denaturation occurs, the detected activity does not change as a function of time. For this reason, the relative activity data change during the assay, and the optimum temperature shifts toward lower values.

Evidence of this shifting balance is that more marked decreases in relative activity were observed in the experiments performed at 42 and 46 °C (Fig 1A), which were the closest temperatures to the *T*_m_ for Sfβgly (45 °C [9]). Moreover, the lower the Sfβgly concentration, the shorter the time required at 42 °C for thermal denaturation to reduce the active enzyme population to a fraction inferior to that present at 37 °C, at which Sfβgly is stable. In fact, with 280 nM Sfβgly, it took 70 min at 42 °C to reduce the active enzyme concentration to a level at which the relative activity was lower than that at 37 °C (Fig 2A), whereas this switching point occurred at 40 min with 140 nM Sfβgly (Fig 1A). Finally, with the lowest protein concentration used (85 nM), the relative activity data for 42 and 37 °C had already exchanged positions at 10 min (Fig 2B).

**Fig 3:**
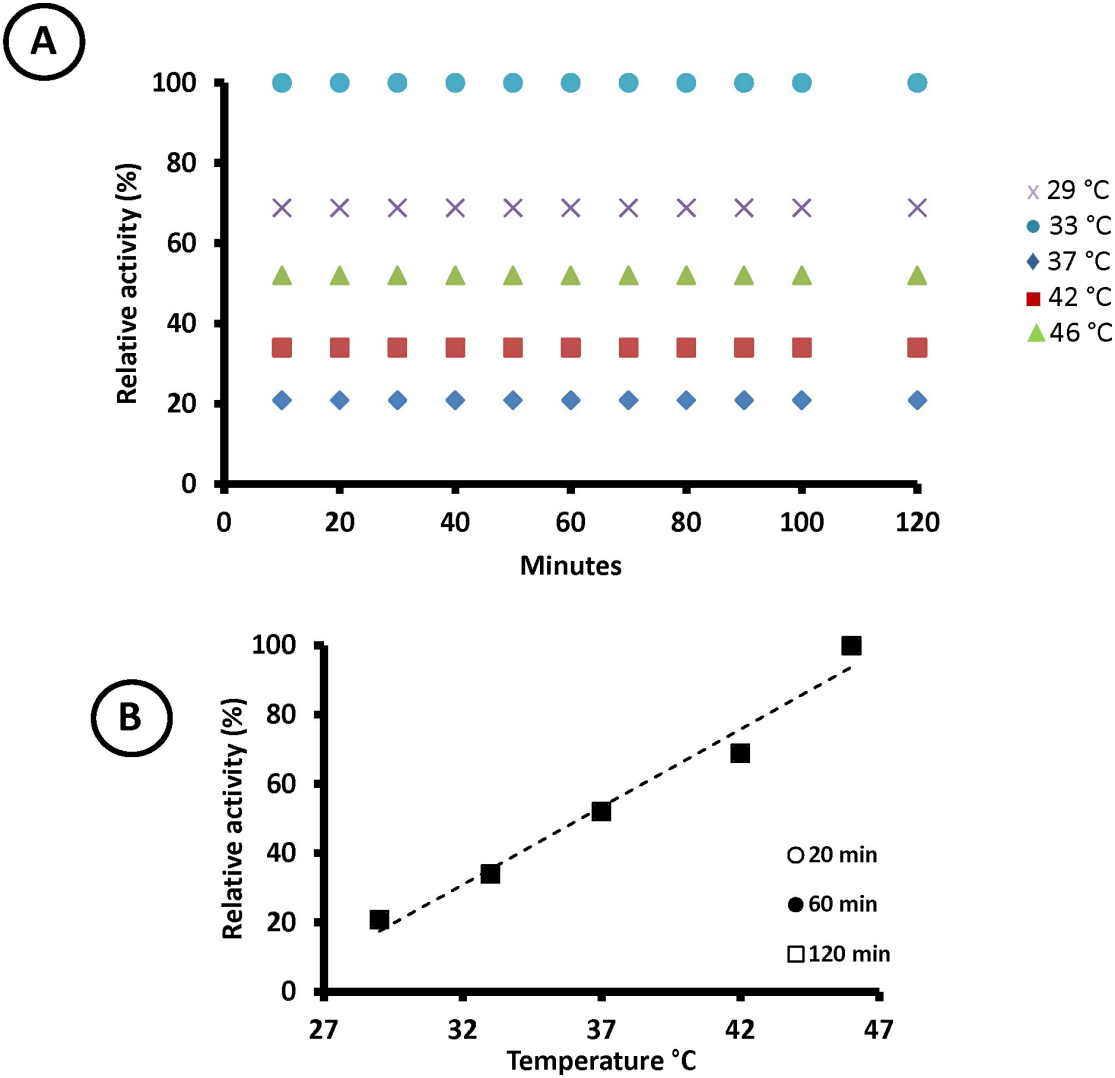
Effect of assay duration on bglTm activity at different temperatures. **A)** Relative activity of bglTm throughout the assay at different temperatures. (purple cross) 29°C; (blue circle) 33°C; (blue diamond) 37°C; (red square) 42°C; (green triangle) 46°C. **B)** Effect of assay time on the relative activity. The enzyme concentration was 7.5 nM. This complete dataset (mean relative activities and respective deviations) is presented on S3 Table.

To determine the mechanism of the optimum temperature variation proposed above, we repeated the same experiments with a thermophilic β-glucosidase, bglTm, from *Thermatoga maritima*, which has a *T*_m_ above 95 °C [10], far higher than 46 °C. Thus, bglTm is stable in the temperature range employed in the experiments, and consequently, the population of active enzyme does not change during the assay. The balance displacements as a function of assay temperature and enzyme *T*_m_ do not apply here, and as expected, the relative activity data (Fig 3A) did not switch positions over time. Moreover, the “optimum temperature plot” shows a continuously increasing line, which resulted exclusively from the temperature effect on the probability that the substrate reached the transition state. This plot did not depend on the assay time (Fig 3B).

Therefore, the classic bell-shaped curves of “optimum temperature plots” are observed only if the enzyme denatures during the activity assay. Thus, one could question whether it is advisable to term the highest point of these plots the “optimum temperature”.

These remarks extend beyond a technical issue. Utilization of the “optimum temperature” for enzyme characterization may result in mistaken kinetic parameters (*K*_m_, *K*_i_ and *k*_cat_) due to the presence of denatured protein in the samples during the initial rate determinations. Importantly, considering its dependence on the assay conditions, the adoption of the “optimum temperature” determined under bench conditions for large-scale uses, which markedly differ in assay duration and enzyme concentration, may result in decreasing reaction rates, lower product yields and financial losses.

## Supporting information

S1 Fig SDS-PAGE analysis of the purified Sfβgly and bglTm. Arrows indicate the purified proteins. The gels were 12% polyacrylamide and proteins were stained with Coomassie Blue R.
(TIFF)

S2 Fig Determination of the enzyme activity at different temperatures. **A**– 280 nM Sfβgly; **B**– 140 nM Sfβgly; **C**– 85 nM Sfβgly; **D**– 7.5 nM bglTm. The enzyme activity was determined based on the slope or the first derivative at each time point. More details are provided in the Material and Methods section.
(TIFF)

S1 Table Relative activity of 140 nM Sfβgly along the assay time at different temperatures. (DOCX)

S2 Table Relative activity of 85 and 280 nM Sfβgly along the assay time at different temperatures. (DOCX)

S3 Table Relative activity of 7.5 nM bglTm along the assay time at different temperatures. (DOCX)
