# Supplementary figures and images for: Optimum temperature may be a misleading parameter in enzyme characterization and application
Source: PLoS One. 2019 Feb 22;14(2):e0212977. doi: 10.1371/journal.pone.0212977 (PMC6386375; doi:10.1371/journal.pone.0212977)

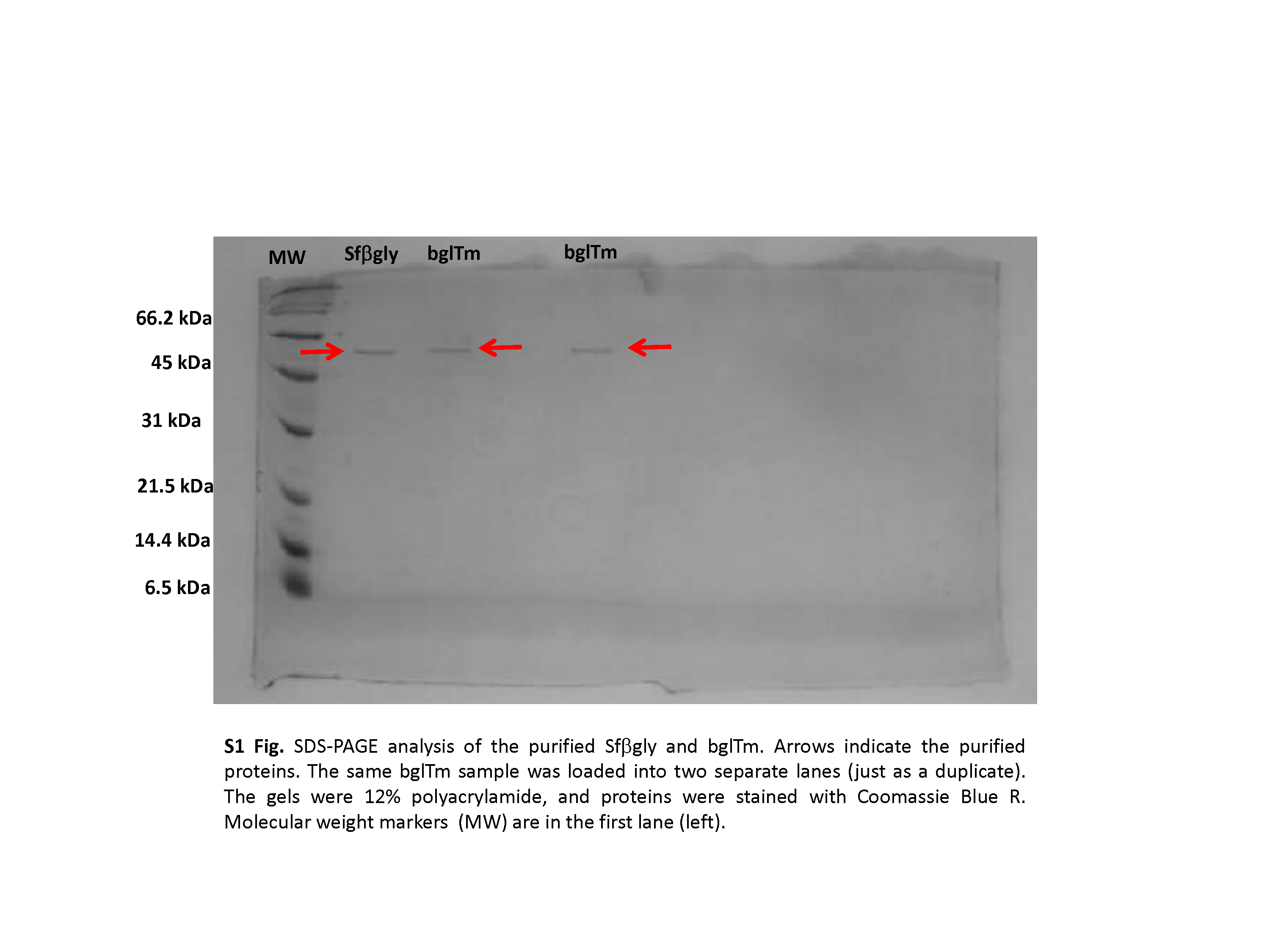

Supplement: S1 Fig — Arrows indicate the purified proteins. The gels were 12% polyacrylamide and proteins were stained with Coomassie Blue R. (TIFF) [file pone.0212977.s001.tiff]

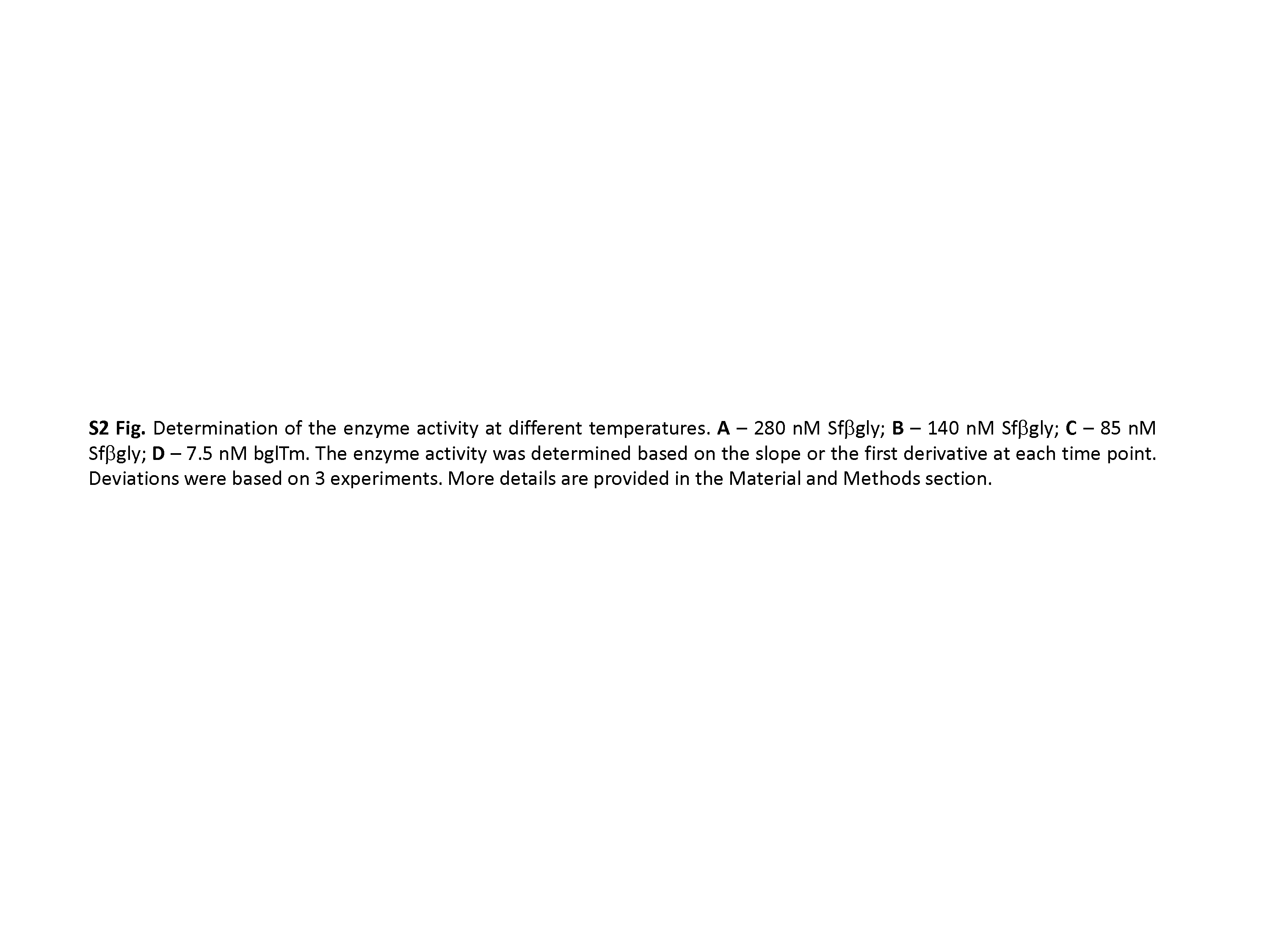

Supplement: S2 Fig — A– 280 nM Sfβgly; B– 140 nM Sfβgly; C– 85 nM Sfβgly; D– 7.5 nM bglTm. The enzyme activity was determined based on the slope or the first derivative at each time point. More details are provided in the Material and Methods section. (TIFF) [file pone.0212977.s002.tiff]
