# Supplementary material for: Optimum temperature may be a misleading parameter in enzyme characterization and application
Source: PLoS One. 2019 Feb 22;14(2):e0212977. doi: 10.1371/journal.pone.0212977 (PMC6386375; doi:10.1371/journal.pone.0212977)
Supplement: S1 Table — (DOCX) [file pone.0212977.s003.docx]

**S1 Table. Relative activity of 140 nM Sfβgly along the assay time at different temperatures**

|  | **Time (min)** | | | | | | | | | | | | | |  |
| --- | --- | --- | --- | --- | --- | --- | --- | --- | --- | --- | --- | --- | --- | --- | --- |
|  | 10 | 20 | 30 | 40 | 50 | 60 | 70 | 80 | 90 | | 100 | | 120 | |  |
| **Temperature ( °C)** | **Relative Activity (%)** | | | | | | | | | | | | | | |
| 29 | 60 ± 6 | 64 ± 7 | 69 ± 7 | 74 ± 7 | 74 ± 7 | 74 ± 7 | 74 ± 7 | 74 ± 7 | | 74 ± 7 | | 74 ± 7 | | 74 ± 7 | |
| 33 | 74 ± 4 | 79 ± 4 | 85 ± 4 | 91 ± 3 | 91 ± 3 | 91 ± 3 | 91 ± 3 | 91 ± 3 | | 91 ± 3 | | 91 ± 3 | | 91 ± 3 | |
| 37 | 81 ± 6 | 87 ± 6 | 94 ± 6 | 100 ± 5 | 100 ± 5 | 100 ± 5 | 100 ± 5 | 100 ± 5 | | 100 ± 5 | | 100 ± 5 | | 100 ± 5 | |
| 42 | 100 ±11 | 100 ± 10 | 100 ± 9 | 99 ± 8 | 92 ± 6 | 86 ± 5 | 80 ± 4 | 74 ± 3 | | 69 ± 2 | | 64 ± 1 | | 55.8 ± 0.6 | |
| 46 | 78 ± 6 | 74 ± 5 | 70 ± 3 | 66 ± 2 | 58 ± 1 | 51 ± 2 | 45 ± 2 | 40 ± 3 | | 35 ± 3 | | 31 ± 3 | | 24 ± 3 | |

Data are the mean ± deviation (n = 3). These data are also presented on Figure 1. Relative activities were calculated based on enzyme assays presented on S2 Fig.
