# Supplementary material for: Optimum temperature may be a misleading parameter in enzyme characterization and application
Source: PLoS One. 2019 Feb 22;14(2):e0212977. doi: 10.1371/journal.pone.0212977 (PMC6386375; doi:10.1371/journal.pone.0212977)
Supplement: S2 Table — (DOCX) [file pone.0212977.s004.docx]

**S2 Table. Relative activity of 280 nM Sfβgly along the assay time at different temperatures**

|  | **Time (min)** | | | | | | | | | | | | | |  |
| --- | --- | --- | --- | --- | --- | --- | --- | --- | --- | --- | --- | --- | --- | --- | --- |
|  | 10 | 20 | 30 | 40 | 50 | 60 | 70 | 80 | 90 | | 100 | | 120 | |  |
| **Temperature ( °C)** | **Relative Activity (%)** | | | | | | | | | | | | | | |
| 29 | 66 ± 3 | 68 ± 3 | 71 ± 4 | 73 ± 4 | 75 ± 4 | 78 ± 4 | 74 ± 3 | 81 ± 5 | | 81 ± 5 | | 81 ± 5 | | 81 ± 5 | |
| 33 | 73 ± 3 | 75 ± 3 | 77 ± 3 | 80 ± 4 | 83 ± 4 | 86 ± 4 | 89 ± 4 | 89 ± 4 | | 89 ± 4 | | 89 ± 4 | | 89 ± 4 | |
| 37 | 81 ± 6 | 84 ± 7 | 86 ± 7 | 89 ± 7 | 92 ± 7 | 96 ± 8 | 99 ± 8 | 100 ± 9 | | 100 ± 9 | | 100 ± 9 | | 100 ± 9 | |
| 42 | 100 ± 0.3 | 100 ± 1 | 100 ± 1 | 100 ± 2 | 100 ± 3 | 100 ± 4 | 100 ± 5 | 97 ± 6 | | 93 ± 7 | | 90 ± 7 | | 84 ± 8 | |
| 46 | 69 ± 4 | 67 ± 4 | 66 ± 3 | 65 ± 3 | 63 ± 3 | 62 ± 3 | 60 ± 2 | 58 ± 2 | | 54 ± 2 | | 51 ± 2 | | 46 ± 1 | |

Data are the mean ± deviation (n = 3). These data are also presented on Figure 2. Relative activities were calculated based on enzyme assays presented on S2 Fig.

**S2 Table. Relative activity of 85 nM Sfβgly along the assay time at different temperatures**

|  | **Time (min)** | | | | | | | | | | | | | |  |
| --- | --- | --- | --- | --- | --- | --- | --- | --- | --- | --- | --- | --- | --- | --- | --- |
|  | 10 | 20 | 30 | 40 | 50 | 60 | 70 | 80 | 90 | | 100 | | 120 | |  |
| **Temperature ( °C)** | **Relative Activity (%)** | | | | | | | | | | | | | | |
| 29 | 66 ± 5 | 66 ± 5 | 66 ± 5 | 66 ± 5 | 66 ± 5 | 66 ± 5 | 66 ± 5 | 66 ± 5 | | 66 ± 5 | | 66 ± 5 | | 66 ± 5 | |
| 33 | 87 ± 8 | 87 ± 8 | 87 ± 8 | 87 ± 8 | 87 ± 8 | 87 ± 8 | 87 ± 8 | 87 ± 8 | | 87 ± 8 | | 87 ± 8 | | 87 ± 8 | |
| 37 | 100 ± 2 | 100 ± 2.8 | 100 ± 2.8 | 100 ± 2.8 | 100 ± 2.8 | 100 ± 2.8 | 100 ± 2.8 | 100 ± 2 | | 100 ± 2 | | 100 ± 2 | | 100 ± 2 | |
| 42 | 89 ± 9 | 87 ± 9 | 85 ± 8 | 83 ± 8 | 81 ± 7 | 80 ± 7 | 78 ± 7 | 76 ± 6 | | 75 ± 6 | | 73 ± 6 | | 70 ± 5 | |
| 46 | 59.1 ± 0.6 | 55.7 ± 0.3 | 52.3 ± 0.1 | 49.2 ± 0.2 | 46.2 ± 0.4 | 43.4 ± 0.5 | 40.7 ± 0.7 | 38.3 ± 0.8 | | 35.9 ± 0.9 | | 33.8 ± 1 | | 30 ± 1 | |

Data are the mean ± deviation (n = 3). These data are also presented on Figure 2. Relative activities were calculated based on enzyme assays presented on S2 Fig.
