# Supplementary material for: Optimum temperature may be a misleading parameter in enzyme characterization and application
Source: PLoS One. 2019 Feb 22;14(2):e0212977. doi: 10.1371/journal.pone.0212977 (PMC6386375; doi:10.1371/journal.pone.0212977)
Supplement: S3 Table — (DOCX) [file pone.0212977.s005.docx]

**S3 Table. Relative activity of 7.5 nM bglTm along the assay time at different temperatures**

|  | **Time (min)** | | | | | | | | | | | |  |
| --- | --- | --- | --- | --- | --- | --- | --- | --- | --- | --- | --- | --- | --- |
|  | 10 | 20 | 30 | 40 | 50 | 60 | 70 | 80 | 90 | | 100 | |  |
| **Temperature ( °C)** | **Relative Activity (%)** | | | | | | | | | | | |  |
| 29 | 23.1 ± 0.8 | 23.1 ± 0.8 | 23.1 ± 0.8 | 23.1 ± 0.8 | 23.1 ± 0.8 | 23.1 ± 0.8 | 23.1 ± 0.8 | 23.1 ± 0.8 | | 23.1 ± 0.8 | | 23.1 ± 0.8 | |
| 33 | 35 ± 1 | 35 ± 1 | 35 ± 1 | 35 ± 1.1 | 35 ± 1 | 35 ± 1 | 35 ± 1 | 35 ± 1 | | 35 ± 1 | | 35 ± 1 | |
| 37 | 52 ± 2 | 52 ± 2 | 52 ± 2 | 52 ± 2 | 52 ± 2 | 52 ± 2 | 52 ± 2 | 52 ± 2 | | 52 ± 2 | | 52 ± 2 | |
| 42 | 68 ± 3 | 68 ± 3 | 68 ± 3 | 68 ± 3 | 68 ± 3 | 68 ± 3 | 68 ± 3 | 68 ± 3 | | 68 ± 3 | | 68 ± 3 | |
| 46 | 100 ± 10 | 100 ± 10 | 100 ± 10 | 100 ± 10 | 100 ± 10 | 100 ± 10 | 100 ± 10 | 100 ± 10 | | 100 ± 10 | | 100 ± 10 | |

Data are the mean ± deviation (n = 3). These data are also presented on Figure 3. Relative activities were calculated based on enzyme assays presented on S2 Fig.
